# Editorial: Insights in renal and epithelial physiology: 2021

**DOI:** 10.3389/fphys.2022.998358

**Published:** 2022-08-29

**Authors:** Carolyn M Ecelbarger, Hui Y Lan

**Affiliations:** ^1^ Department of Medicine/Division of Endocrinology and Metabolism, Georgetown University, Washington, DC, United States; ^2^ Department of Medicine and Therapeutics, The Chinese University of Hong Kong, Hong Kong, China

**Keywords:** kidney, transport, potassium, fibrosis, therapeutics

We were delighted to host this Research Topic on current newer insights in renal physiology. We were not disappointed with the high-quality yield. The kidney is premier in whole-body chemical homeostasis. The renal proximal tubule (PT) is the major site for bulk reabsorption of a variety of substances. Conservation of Fe^2+/3+^, a transition metal, is critical for life, due to its essential role in oxygen transport as a component of hemoglobin, as well as, a constituent of other enzymes, e.g., cytochromes (Ponka, 1999). Although Fe^2+/3+^ transporters have been reported to be expressed in renal tubule (Thevenod and Wolff, 2016), including DMT1 (divalent metal transporter 1), ZIP8 (zinc, iron transporter 8) and ZIP14 (zinc, iron transporter 14), the understanding surrounding the regulation of filtered Fe^2+/3+^ remains limited (Wareing and Smith). (Frolkis et al., 2010) provided one of our first glimpses of quantitative assessment of renal PT Fe^2+/3+^ reabsorption. Using the classic approach of micropuncture of the S2 segment of the PT from male Wistar rats, the authors were able to determine that, on the whole, Fe^2+/3+^ is reabsorbed (rather than secreted or unaffected) by the PT. They found that about 1/3rd of filtered Fe^2+/3+^ was reabsorbed and calculated, for the first time, a Fe^2+/3+^ transport rate for renal PT S2. The investigators went on to model renal Fe^2+/3+^ handling, and concluded that without renal reabsorption the total amount of plasma Fe^2+/3+^ would be depleted within 2 h (in the rat). Additional studies will be needed to determine the identity of transport proteins involved.

Due to its high energy demands for transport, the PT, especially the S3 segment, is highly susceptible to injury (Vallon, 2011). Chen et al. provide a comprehensive review nicely summarizing the condition of high-fat-diet induced renal inflammatory injury in chronic kidney disease (CKD). They begin by emphasizing the high world-wide prevalence of CKD (at about 9.1% of the population) (Bikbov et al., 2020). This was followed by some discussion of how dietary intake of fat has increased. The review provides a satisfying discussion of potential mechanisms whereby increased lipid accumulation in the cells of the PT can result in cellular injury and death via a number of potential inflammatory pathways.

One of the “chemicals” carefully balanced by the kidney is potassium (K^+^) (Palygin et al., 2017). Epilepsy, ataxia, sensorineural deafness, and renal tubulopathy (EAST), also known as, seizures, sensorineural deafness, ataxia, intellectual disability, and electrolyte imbalance (SESAME) Syndrome is a disorder associated with mutations in the inwardly-rectifying potassium (K^+^) channel, Kir4.1 (KCNJ10) (Paulais et al., 2011). Kir5.1 (KCNJ16), a similar channel, which heteromerizes with Kir4.1 in kidney to form functional tetrameric channels, can also be mutated. This review provides a comprehensive discussion of the function, tissue localization, and impact of mutations in these two inwardly-rectifying K^+^ channels. They discuss how mutations relate to impaired K^+^ clearance from various intra- and extracellular spaces. They also highlight cellular mechanisms determining the difference in phenotypes observed when Kir4.1 versus Kir5.1 is mutated.

The post-macula densa portion of the renal tubule is key in whole-body K^+^ handling. Zapf et al. provide a novel and intriguing study of how low-salt delivery to the distal nephron triggers the autocrine release of prostaglandin E2 (PGE2) in familial hyperkalemic hypertension (FHHt) mice. The investigators utilize their mouse model of FHHt in which they constitutively activate the STE20 proline alanine-rich kinase (SPAK), which activates the thiazide-sensitive NaCl cotransporter (NCC) (Nguyen et al., 2013). The increase in NCC activity in the distal convoluted tubule reduces the Na^+^ load to the “aldosterone-sensitive distal nephron,” including the connecting tubule and collecting ducts (CD). The study uncovers a role for increased synthesis of PGE2 under low-salt conditions (supported by additional studies *in vitro*) in helping to restore K^+^ balance in these mice by rescuing renal outer medullary potassium channel (ROMK) activity. Overall, the article is a delight for integrative physiologists interested in how different parts of the renal tubule work together to manage electrolyte balance. This work also lends credence to the possibility of manipulating PGE2 or its receptor EP1 in hyperkalemic states, such as in chronic kidney disease (CKD) as a therapeutic strategy.

Yang et al., examine the role of glucocorticoid regulation of vasopressin responses in kidney CD cells. The study uses transcriptomics to evaluate the impact of glucocorticoid receptor knockdown in mpkCCD cells and found that 3,785 upregulated transcripts are associated with 42 KEGG pathways including tumor necrosis factor (TNF) and transforming growth factor, beta (TGFβ) signaling. One main mechanism whereby glucocorticoids appear to enhance the actions of vasopressin is by increasing the expression of the V2 receptor. Overall, this report provided novel insights into inter-relationships between these hormonal systems. Down-regulation of glucocorticoid receptors also bear some resemblance to the underlying changes in the CD found in vasopressin escape (VPE), including reduced V2 receptor (Tian et al., 2004) and activation of TNF and TGFβ signaling (Lee et al., 2018). Whether reduced glucocorticoids play any role in augmenting VPE is unclear; however, we found aldosterone levels increased in a rat model of VPE (Song et al., 2004). Further exploration of these interactions is warranted.

One does not often consider the potential side effects of doxycycline-induced genetic recombination (a commonly used tool to change gene expression). Jung et al. found that addition of DOX changed the abundance of 1,549 transcripts at 3 days and 2,643 transcripts at 6 days in mIMCD3 cells grown on permeable supports. This report finds a number of pathways altered; most prominently, they find DOX reduced transcripts related to cell proliferation. However, how long the pattern of transcriptomic changes exists and its reversibility is still in need of study.

Fibrosis in the kidney can be initiated by factors other than inflammation (Rohatgi and Flores, 2010). Xie et al. examined the role of mechanical stretch in triggering the epithelial-mesenchymal transition in keratinocytes. They uncover a role for the Piezo1 channel, which has been reported to play a role in a number of cell types, especially epithelial, detecting “stretch” and translating this into adaptive cellular changes, e.g., proliferation. Using human primary epidermal keratinocytes (HEKs), the investigators showed mechanical stretch increased calcium influx. Knockdown of Piezo1 (siRNA) or inhibition of the channel with GsMTx4 attenuated calcium influx, supporting a role for Piezo in mechanically-induced calcium influx. They also showed similar maneuvers altered HEK morphology and expression of epithelial-to-mesenchymal transition (EMT) markers. Overall, these authors appeared to have discovered a mechanistic link between mechanical stress and EMT, i.e., Piezo1. Thus Piezo1 may be a novel anti-fibrotic target, and its role in other cell types should be explored.

In sum, we thank all the authors. The year 2021, despite being a difficult time for many of us, provided a number of novel insights in the field of renal physiology and pathophysiology. Spanning the range from fundamental and technical advances to reviews relating to clinical disorders and the kidney, this Research Topic did not disappoint.
